# Potential plasma biomarkers at low altitude for prediction of acute mountain sickness

**DOI:** 10.3389/fimmu.2023.1237465

**Published:** 2023-09-28

**Authors:** Haoran Guo, Qi Wang, Tao Li, Jingwen Chen, Chao Zhang, Ying Xu, Qing Chang, Hangyi Li, Weiqiang Sun, Ruidi Han, Chi Wang, Chengbin Wang

**Affiliations:** ^1^Medical School of Chinese PLA, Beijing, China; ^2^Department of Laboratory Medicine, The First Medical Center of Chinese PLA General Hospital, Beijing, China; ^3^Department of Orthopeadics, The Fourth Medical Center of Chinese PLA General Hospital, Beijing, China; ^4^Outpatient Department of Chinese People's Liberation Army No. 69316 Troops, Xinjiang, China; ^5^Department of Hyperbaric Chamber, The First Medical Center of Chinese PLA General Hospital, Beijing, China; ^6^Xinjiang Hotan Military Subdistrict, Xinjiang, China

**Keywords:** acute mountain sickness, susceptibility, proteomics, biomarkers, LC-MS/MS

## Abstract

**Background:**

Ascending to high altitude can induce a range of physiological and molecular alterations, rendering a proportion of lowlanders unacclimatized. The prediction of acute mountain sickness (AMS) prior to ascent to high altitude remains elusive.

**Methods:**

A total of 40 participants were enrolled for our study in the discovery cohort, and plasma samples were collected from all individuals. The subjects were divided into severe AMS-susceptible (sAMS) group, moderate AMS-susceptible (mAMS) group and non-AMS group based on the Lake Louise Score (LLS) at both 5000m and 3700m. Proteomic analysis was conducted on a cohort of 40 individuals to elucidate differentially expressed proteins (DEPs) and associated pathways between AMS-susceptible group and AMS-resistant group at low altitude (1400m) and middle high-altitude (3700m). Subsequently, a validation cohort consisting of 118 individuals was enrolled. The plasma concentration of selected DEPs were quantified using ELISA. Comparative analyses of DEPs among different groups in validation cohort were performed, followed by Receiver Operating Characteristic (ROC) analysis to evaluate the predictive efficiency of DEPs for the occurrence of AMS.

**Results:**

The occurrence of the AMS symptoms and LLS differed significantly among the three groups in the discovery cohort (*p*<0.05), as well as in the validation cohort. Comparison of plasma protein profiles using GO analysis revealed that DEPs were primarily enriched in granulocyte activation, neutrophil mediated immunity, and humoral immune response. The comparison of potential biomarkers between the sAMS group and non-AMS group at low altitude revealed statistically higher levels of AAT, SAP and LTF in sAMS group (*p*=0.01), with a combined area under the curve(AUC) of 0.965. Compared to the mAMS group at low altitude, both SAP and LTF were found to be significantly elevated in the sAMS group, with a combined AUC of 0.887. HSP90-α and SAP exhibited statistically higher levels in the mAMS group compared to the non-AMS group at low altitude, with a combined AUC of 0.874.

**Conclusion:**

Inflammatory and immune related biological processes were significantly different between AMS-susceptible and AMS-resistant groups at low altitude and middle high-altitude. SAP, AAT, LTF and HSP90-α were considered as potential biomarkers at low altitude for the prediction of AMS.

## 1 Introduction

In recent years, there has been a growing trend of individuals venturing to high altitudes for the purpose of tourism, sport activities, work or military tasks. Extensive research has been conducted on the physiological variations resulting from high-altitude exposure in the context of hypobaric hypoxia for several decades. Inadaptation to high altitude can give rise to a spectrum of conditions ranging from acute mountain sickness (AMS) to severe ailments such as high-altitude pulmonary edema (HAPE) and high-altitude cerebral edema (HACE). The occurrence of AMS has been reported in over 25% of individuals at the altitude of 3500 m and more than 50% of individuals at the altitude of 6000 m (1), significantly impacting critical military operations conducted at high altitudes. However, researchers have observed variations in the adaptive capacity to high altitudes among individuals accustomed to lower altitudes (2, 3). Therefore, it is of paramount importance to identify susceptible individuals at low altitude who could not adapt themselves following rapid ascent to high altitude. These individuals should either avoid rapid ascents or receive preventative treatment prior to ascending.

Liquid chromatography-mass spectrometry (LC/MS) is widely employed in the proteomic field to explore potential biomarkers, and elucidate the underlying biological mechanisms of various diseases. Previous proteomic studies identified biomarkers and elucidated mechanisms between AMS-susceptible group and AMS-resistant group through proteomic analysis at high altitude (3–5), revealing a significant upregulation of proteins with antioxidant properties in AMS-susceptible individuals (4). In addition, Yang et al. identified ADAM15 and TRAF2 as protective and diagnostic biomarkers of AMS, respectively (3). It has also been reported that acute exposure to high altitude can induce alterations in immunological indices (6). Regulation of inflammation and response to hypoxia have been reported to exhibit significant crosstalk in terms of signaling pathways, potentially playing crucial roles in the physiological response to hypobaric hypoxia (7, 8). Exposure to hypoxia at high altitude leads to altered inflammatory characteristics, which may contribute significantly to adaptation (9). Researchers also utilized microarray data from peripheral blood mononuclear cell (PBMC) or identified circulating microRNAs to predict AMS (10, 11). But few studies focus on exploring the plasma proteome at low altitude grouped by whether AMS occurs at different high altitudes.

Thus, in our current study, we recruit volunteers who ascend to extreme high-altitude (5000m) after a sojourn at middle high-altitude (3700m), which represents an innovative approach not previously reported in the field. This ascending protocol is particularly suitable for low altitude populations acclimatizing to enter high altitude, especially for those exceeding 5000m. Through proteomic analysis of plasma using mass spectrometry at low altitude and middle high-altitude, we hope to distinguish the individuals who could adapt to high altitude from those who are not suitable for exposure to high altitude before their ascending.

## 2 Materials and methods

### 2.1 Study population

Our study recruited 40 and 118 Chinese male volunteers who primarily lived at low altitude as discovery cohort and validation cohort respectively. The inclusion criteria were as follows: individuals enrolled should not have acute infection and reside at low altitude during the past 6 months, with no chronic inflammatory diseases, and with the age above 18 years. Volunteers were excluded if they had any health problems, abnormal blood routine test results or biochemical tests, or had traveled to high altitude within the past 6 months. The general health eligibility of these enrolled volunteers including medical history, physical examination, blood and urine routine tests were assessed prior to their participation. All enrolled participants have signed informed consent. This study was approved by the Ethic Committee of Chinese PLA General Hospital (approval identifier: S2021-623-01) and all protocols followed the ethical guidelines.

### 2.2 Experiment design

Before entering the high altitude area, all the enrolled volunteers completed the questionnaire at the altitude of 1400m (Kashgar, China). Demographic information including age, weight, height and smoking habits were obtained. Blood samples at low altitude were collected in the morning as the baseline data. Subsequently, these individuals travelled by car to the altitude of 3700 m. Following a seven-day adaptation at 3700m, they further ascended to the extreme altitude of 5000 m within 10 hours’ tour by car. After their ascent to altitudes of 3700m and 5000m (36-48 hours), fasting blood samples were collected from all participants, along with completion of a questionnaire based on 2018 Lake Louise scoring (12). AMS was defined as a participant having a Lake Louise score (LLS) was above 3, indicating symptoms such as headache, fatigue and/or weakness, dizziness/light-headedness and gastrointestinal symptoms. It was mandatory for individuals diagnosed with AMS to experience at least one point from headache during ascent to the high altitude. The specific scoring criteria for Lake Louise Acute Mountain Sickness (LLS) in 2018 were presented in **Supplementary Table 1**. Subjects were categorized into AMS group or non-AMS group according to the LLS at different high altitudes, namely middle high-altitude of 3700m and extreme high-altitude of 5000m. There were two sub-groups in AMS group. Subjects developing AMS at the altitude of 3700m, as well as 5000m, were considered as severe AMS-susceptible (sAMS) group. Subjects remaining resistant at the altitude of 3700m, but developing AMS at the altitude of 5000m, were considered as moderate AMS-susceptible (mAMS) group.

### 2.3 Preparation of plasma samples for proteome analysis

Supernatant was collected by centrifuging plasma samples at 3000 rpm. Samples were then transported to our laboratory in Beijing and stored under −80°C until mass spectrometry (MS) analysis was conducted. To mitigate interference from high-abundance proteins on the identification of low-abundance proteins, we employed the Top14 Abundant Protein Depletion Mini Spin Columns (Thermo Fisher, USA). For each sample, proteins were re-solubilized using a 10kD filter-aided sample preparation (FASP) tube (UFC501024, Millipore, USA) with 200μl of 8mmol/L urea. The filtrate was discarded after centrifugation at 14,000g for 10 minutes. Subsequently, the samples were treated with dithiothreitol (Sigma, USA) and iodoacetamide (Sigma, USA), followed by digestion into peptides using trypsin (Promega, USA) according to the previously published methods (13). Finally, the desiccated peptides were resuspended in 0.1% formic acid for LC-MS/MS proteome analysis.

### 2.4 LC-MS analysis

The plasma proteomics analysis was conducted using the EASY-nLC 1200 nano system in conjunction with an Orbitrap Exploris 480 mass spectrometer (Thermo Scientific, San Jose, CA). The mass spectrometer was operated in positive mode with the FAIMS Pro interface. Data-dependent acquisition (DDA) was performed employing a 75-minute gradient and the following parameters (14). An electrospray voltage of 2.2 kV was applied and a scan range from 350 to 1,500 m/z was set. Compensation voltages of -45 or -65 V were utilized to achieve accurate mass determination. The resolutions for MS1 and MS2 were set at 60,000 and 15,000, respectively, with a normalized automatic gain control (AGC) target of 300% and 75%. The maximum injection time was set to 50 ms for MS1, while for MS2 it was reduced to 22 ms. An isolation window of 1.6 m/z was employed along with a normalized collision energy of 27%.

### 2.5 Data processing and bioinformatic analysis

The Q-Exactive raw files were analyzed using Thermo Proteome Discoverer (version 2.1) with the Sequest HT search algorithm and data was identified against the Uniprot human database. Preprocessing of raw data for post-sum normalization, logarithmic transformation and normal distribution imputation was performed using Perseus. Proteins with a false discovery rate (FDR) < 1% and a minimum score for peptides > 40 were considered, while those with missing value > 80% in each group were excluded. Differentially expressed proteins (DEPs) were identified using a two-tailed Student’s test with *q* values (FDR corrected *p* value) <0.05 and fold change > 1.5 or < 0.67. As for the screened DEPs between arbitrary two groups, heatmaps were performed using Oebiotech online tool (https://cloud.oebiotech.cn), volcano plots, partial least squares discriminant analysis (PLS-DA), Gene ontology (GO) analysis and Kyoto encyclopedia of genes and genomes (KEGG) database evaluation were conducted using the R software. “Mfuzz” package was performed to obtain expression pattern clustering according to the expression levels of all potential DEPs among three groups at low altitude.

### 2.6 Validation of selected proteins expression

Four DEPs, heat shock protein HSP 90-alpha(HSP90-α), serum amyloid P-component(SAP), alpha-1-antitrypsin(AAT), and lactotransferrin(LTF), were selected to validate the proteomic results. The concentration of these proteins in plasma were measured using ELISA. The ELISA kits of these proteins were purchased from Fine Test (Wuhan, China) and abcam (USA) and were used according to the manufacturer’s instructions.

As for ELISA kits from Fine Test, samples with specific dilution ratio were added and incubated for 90 minutes at 37°C. The liquid was removed and wells were washed. The biotinylated antibody (1x) was added to each well with incubation for 60 minutes at 37°C. Then the liquid was removed and wells were washed. The horse radish peroxidase(HRP)‐antibody (1×) was added to each well with incubation for 30 minutes at 37°C. Subsequently, TMB was added and incubated for 10-20 minutes at 37°C away from light. Finally, stop solution was added and the optical density was detected at 450 nm immediately using microplate reader.

### 2.7 Statistical analysis

The normality of all data was evaluated via the Kolmogorov–Smirnov test or Shapiro-Wilk test. Mean ± standard deviation (SD) was used for the normally distributed data, while the median [25–75% interquartile range (IQR)] was used for non-normally distributed continuous data. Comparisons between two groups were evaluated by t-tests, while among three groups were evaluated by one way analysis of variance (ANOVA) for normally distributed data. Non-normally distributed continuous data was evaluated by Mann-Whitney *U* tests or Kruskal-Wallis H test between different groups. Categorical variables were presented as ratio and evaluated by chi-square tests. Receiver operating characteristic (ROC) analysis, which generate parameters including area under the ROC(AUC), sensitivity, and specificity were performed to evaluate the predictive performance of biomarkers. All statistical analyses were performed using SPSS statistical software 26.0 and GraphPad Prism 9 software. *P* < 0.05 was considered statistically significant.

## 3 Results

### 3.1 AMS status and general information of participants in discovery and validation cohorts

**Figure 1:**
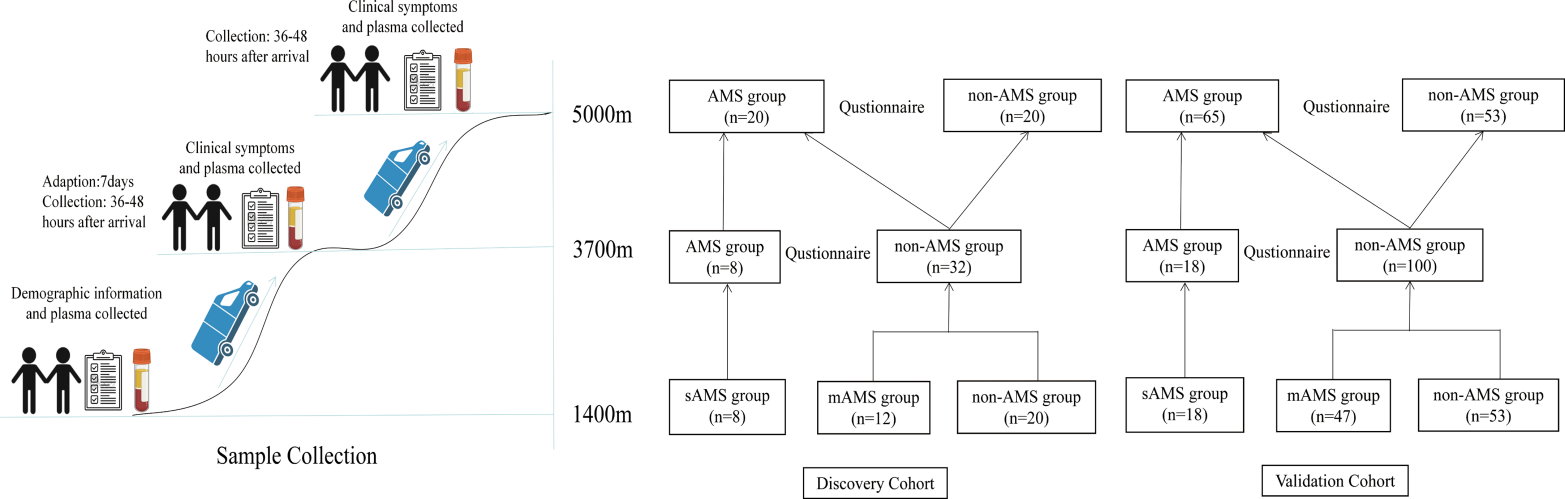
Flowchart of the study design.

Among the enrolled 40 individuals, 8 subjects developed AMS and the remaining 40 subjects were negative for AMS at the altitude of 3700m. Then after further ascending to the altitude of 5000m, these 8 subjects were still AMS, while 12 subjects among the 40 subjects developed AMS, leaving the other 20 still as non-AMS. Thus, based on the LLS at both the altitude of 3700m and 5000m, we considered these 8 subjects who developed AMS at middle high-altitude (3700m) as sAMS group, while the other 12 subjects who developed AMS at extreme high-altitude (5000m) but remained resistant at middle high-altitude (3700m) as mAMS group. The other 20 subjects who remained negative for AMS at both 3700m and 5000m were considered as AMS resistant, which was named as non-AMS group in our study. The study design is showed in **Figure 1**.

There were no statistical differences in age, weight, height, BMI and smoking between AMS group and non-AMS group of discovery cohort both at the altitude of 5000m and 3700 m (**Supplementary Tables S2, S3**). The occurrence proportion of the AMS symptoms including headache, gastrointestinal upset, fatigue, dizziness, and LLS in AMS group were higher than non-AMS group both at 5000m and 3700m (*p*<0.05). The occurrence proportion of insomnia, the symptom excluded by 2018 Lake Louise Acute Mountain Sickness score, differed between AMS group and non-AMS group at 5000m, but not at 3700m (**Supplementary Tables S2, S3**). Also, there were no statistical differences in age, weight, height, BMI and smoking among sAMS group, mAMS group and non-AMS group. The occurrence proportion of the AMS symptoms and LLS among the three groups were statistically different (*p*<0.05) (**Table 1**). Among the three groups, LLS was statistically different between sAMS group and non-AMS group, as well as between mAMS group and non-AMS group (*p*<0.05).

**Table 1:** Demographic information, AMS symptoms and LLS of discovery cohort divided in the three groups at altitude of 5000m.

	sAMS group (n=8)	mAMS group (n=12)	Non-AMS group (n=20)	*P*
Age	23.5(22.25 , 27.75)	24(22.25 , 26)	22(21 , 25)	0.243
Weight(kg)	71.75(8.77903)	68.1667(5.74984)	69.3(6.0793)	0.493
Height(cm)	177.375(3.81491)	173.6667(5.88269)	175.05(3.89973)	0.218
BMI	22.7957(2.57397)	22.5868(1.24678)	22.636(2.1183)	0.973
Smoking	50%(4/8)	70.59%(12/17)	60.87%(14/23)	0.622
Headache	100%(8/8)	100%(12/12)	35%(7/20)	<0.001
Gastrointestinal upset	75%(6/8)	75%(9/12)	30%(6/20)	0.018
Fatigue	100%(8/8)	100%(12/12)	65%(13/20)	0.011
Dizziness	87.5%(7/8)	100%(12/12)	45%(9/20)	0.001
Insomnia	100%(8/8)	100%(12/12)	45%(9/20)	<0.001
LLS	4.5(3.25 , 6)	5(4 , 5)	2(1.25 , 2)	<0.001

There were no statistical differences in age, weight, height, BMI and smoking among sAMS, mAMS and non-AMS groups in validation cohort (**Table 2**). The occurrence proportion of the AMS symptoms and LLS among the three groups were statistically different (*p*<0.05) (**Table 2**). Among the three groups, LLS was statistically different between sAMS group and non-AMS group, as well as between mAMS group and non-AMS group (*p*<0.05).

**Table 2:** Demographic information, AMS symptoms and LLS of validation cohort divided in the three groups at altitude of 5000m.

	sAMS group (n=18)	mAMS group (n=47)	Non-AMS group (n=53)	*P*
Age	22.5(21,24)	22(21,23)	22(21,23)	0.636
Weight(kg)	68.7222(4.9682)	65.4149(7.41203)	66.1698(6.27184)	0.197
Height(cm)	175(170.75,178.25)	175(170,178)	173(170,178)	0.702
BMI	22.5188(1.70433)	21.5156(2.01238)	22.0519(2.06138)	0.155
Smoking	66.7%(12/18)	78.7%(37/47)	66% (35/53)	0.339
Headache	100%(18/18)	100%(47/47)	24.5%(13/53)	<0.001
Gastrointestinal upset	72.2%(13/18)	53.2%(25/47)	11.3%(6/53)	<0.001
Fatigue	100%(18/18)	93.6%(44/47)	32.1%(17/53)	<0.001
Dizziness	88.9%(16/18)	89.4%(42/47)	26.4%(14/53)	<0.001
Insomnia	88.9%(16/18)	78.7%(37/47)	39.6%(21/53)	<0.001
LLS	4.5(3.75,6)	3(3,4)	1(0,2)	0.001

### 3.2 Comparisons of plasma protein profiles among sAMS group, mAMS group and non-AMS group at low altitude in discovery cohort

**Figure 2:**
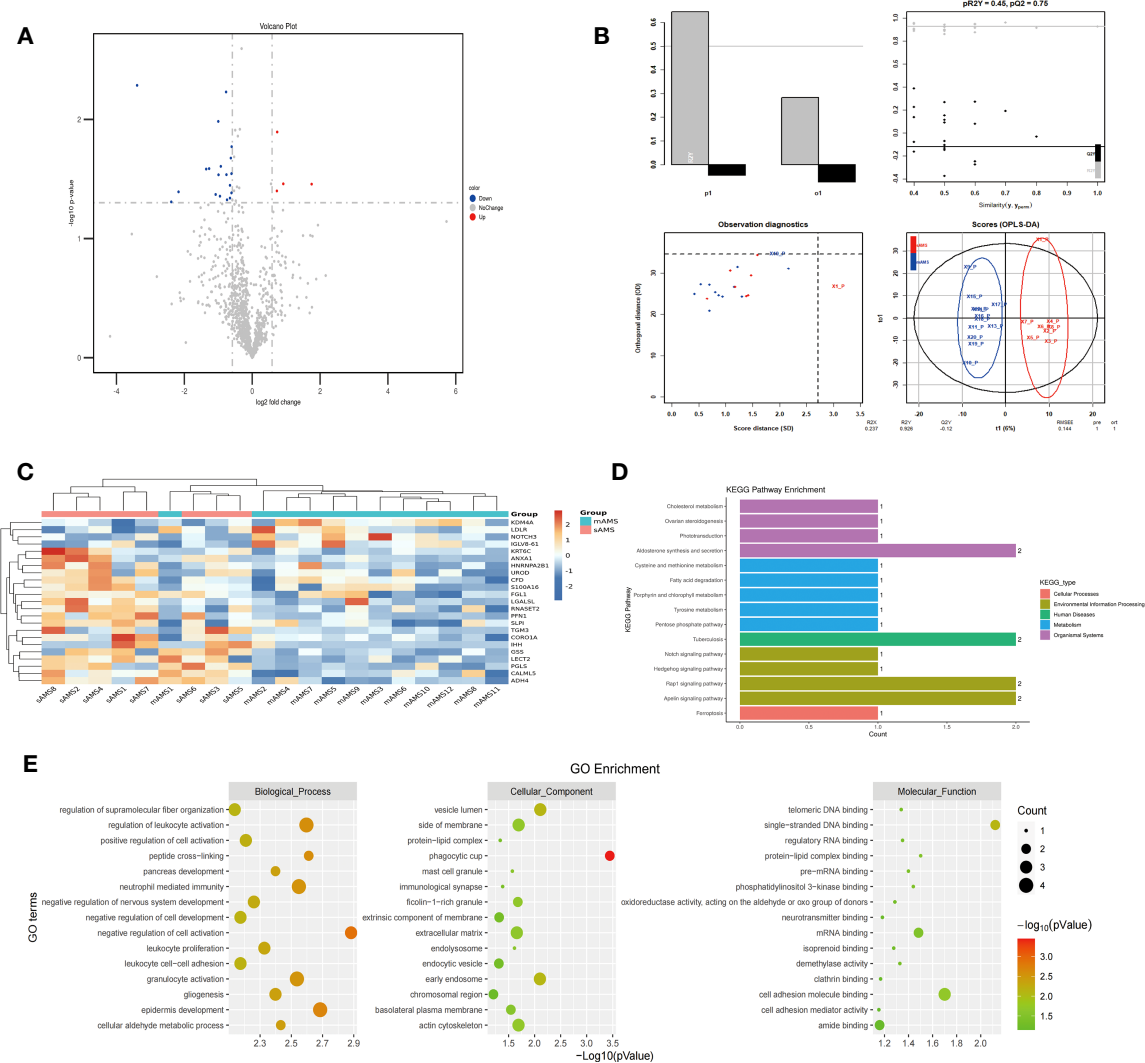
Comparison of plasma differential protein profiles between sAMS group and mAMS group at low altitude. **(A)** Volcano plots show the distribution of DEPs between sAMS group and mAMS group at low altitude. **(B)** PLS-DA plots show clear separation between the sAMS and mAMS groups at low altitude. **(C)** Heatmap represents gene expression trends in sAMS and mAMS groups at low altitude. **(D)** KEGG enrichment shows the canonical pathways of DEPs. **(E)** GO enrichment shows the top fifteen terms in the biological process, cellular component, and molecular function categories between sAMS and mAMS groups at low altitude.

The comparison of plasma protein profiling between 8 sAMS individuals and 12 mAMS individuals revealed a total of 23 DEPs, including 4 up-regulated proteins and 19 down-regulated proteins (**Figure 2A**). Heatmap and PLS-DA analysis revealed clear classification of sAMS and mAMS at low altitude (**Figures 2B, C**). GO analysis revealed that the DEPs were mainly enriched in the cellular components including phagocytic cup, vesicle lumen and side of membrane, biological processes including neutrophil mediated immunity and granulocyte activation, molecular functions including single-stranded DNA binding, cell adhesion molecule binding and mRNA binding. KEGG analysis revealed that the enriched pathways mainly included aldosterone synthesis and secretion, tuberculosis and rap1 signaling pathway (**Figures 2D, E**).

**Figure 3:**
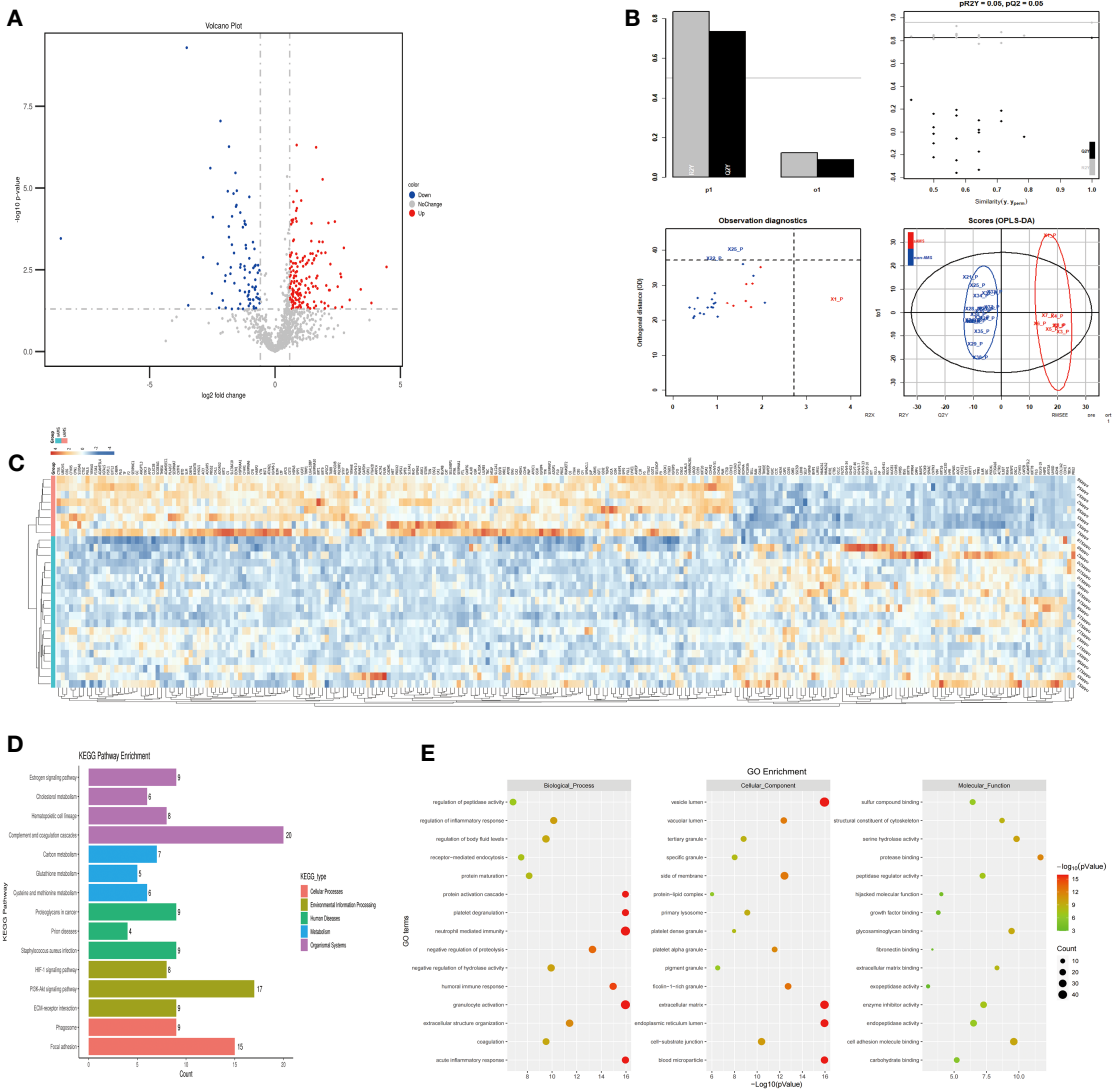
Comparison of plasma differential protein profiles between sAMS group and non-AMS group at low altitude. **(A)** Volcano plots show the distribution of DEPs between sAMS group and non-AMS group at low altitude. **(B)** PLS-DA plots show clear separation between the sAMS and non-AMS groups at low altitude. **(C)** Heatmap represents gene expression trends in sAMS and non-AMS groups at low altitude. **(D)** KEGG enrichment shows the canonical pathways of DEPs. **(E)** GO enrichment shows the top fifteen terms in the biological process, cellular component, and molecular function categories between sAMS and non-AMS groups at low altitude.

The plasma protein profiling of 8 individuals with sAMS and 20 individuals with non-AMS revealed a total of 248 differential proteins, including 164 up-regulated proteins and 84 down-regulated proteins (**Figure 3A**). PLS-DA analysis showed that these proteins clearly distinguished sAMS and nAMS groups and a clustering analysis also showed clear separation between these two groups at low altitude (**Figures 3B, C**). By performing a functional enrichment analysis of the DEPs, we identified cellular components including vesicle lumen, side of membrane and vacuolar lumen, biological processes including neutrophil mediated immunity and platelet degranulation, molecular functions including protease binding and cell adhesion molecule binding, KEGG pathways including complement and coagulation cascades, and PI3K-Akt signaling pathway (**Figures 3D, E**).

**Figure 4:**
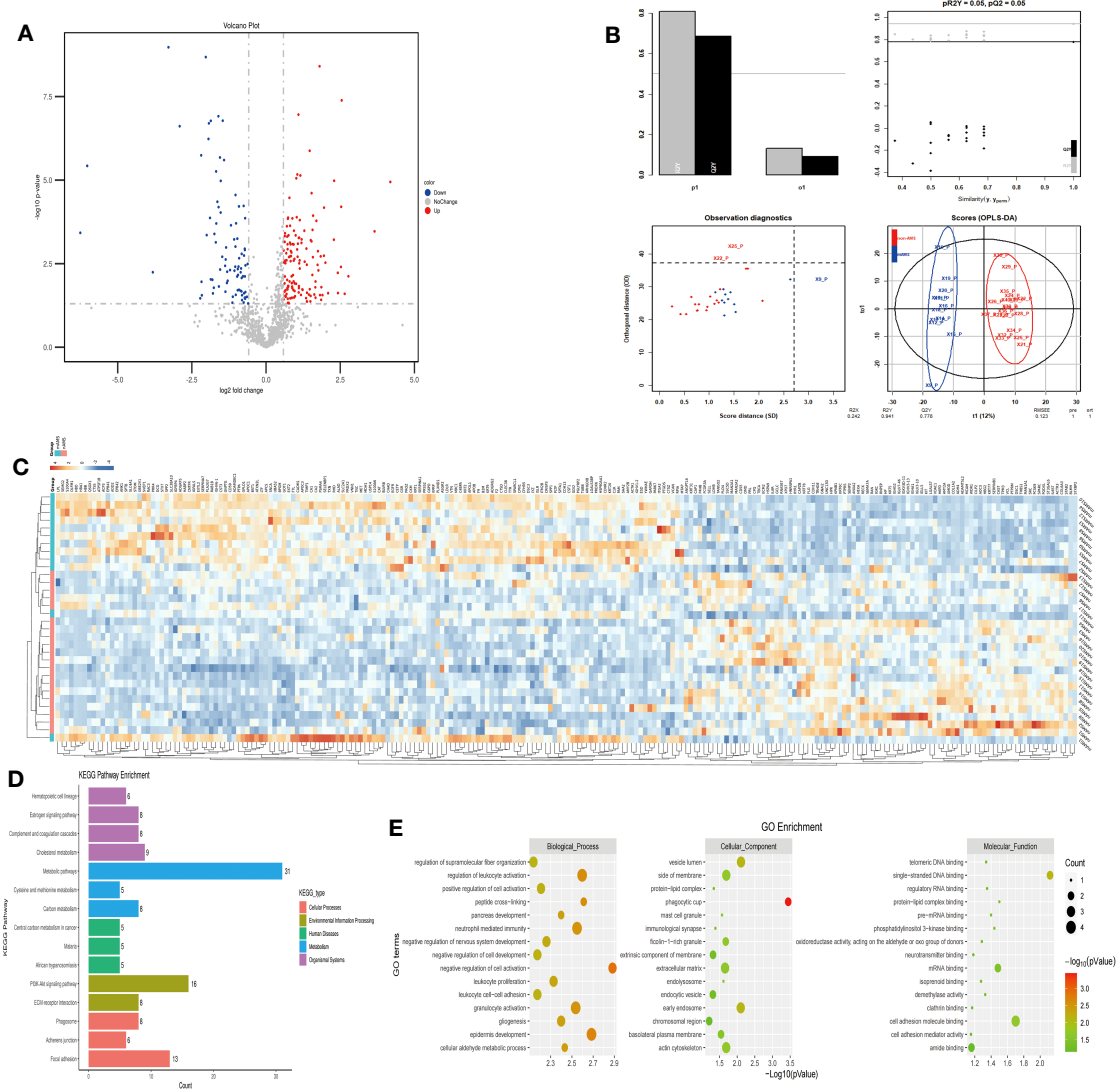
Comparison of plasma differential protein profiles between mAMS group and non-AMS group at low altitude. **(A)** Volcano plots show the distribution of DEPs between mAMS group and non-AMS group at low altitude. **(B)** PLS-DA plots show clear separation between the mAMS and non-AMS groups at low altitude. **(C)** Heatmap represents gene expression trends in mAMS and non-AMS groups at low altitude. **(D)** KEGG enrichment shows the canonical pathways of DEPs. **(E)** GO enrichment shows the top fifteen terms in the biological process, cellular component, and molecular function categories between mAMS and non-AMS groups at low altitude.

The plasma protein profiling of 12 individuals with mAMS and 20 individuals with non-AMS revealed a total of 228 differential proteins, including 140 up-regulated proteins and 88 down-regulated proteins (**Figure 4A**). Heatmap and PLS-DA analysis revealed clear classification of mAMS and non-AMS at the altitude of 1400m (**Figures 4B, C**). GO analysis revealed that the DEPs were mainly associated with the cellular components including vesicle lumen, side of membrane and vacuolar lumen, biological processes including neutrophil mediated immunity, humoral immune response, and granulocyte activation, molecular functions including structural constituent of cytoskeleton and cell adhesion molecule binding. KEGG analysis revealed that the enriched pathways mainly included complement and coagulation cascades, metabolic pathways and PI3K-Akt signaling pathway (**Figures 4D, E**).

### 3.3 Comparison of the plasma protein profiles between mAMS and non-AMS group at the altitude of 3700m in discovery cohort

**Figure 5:**
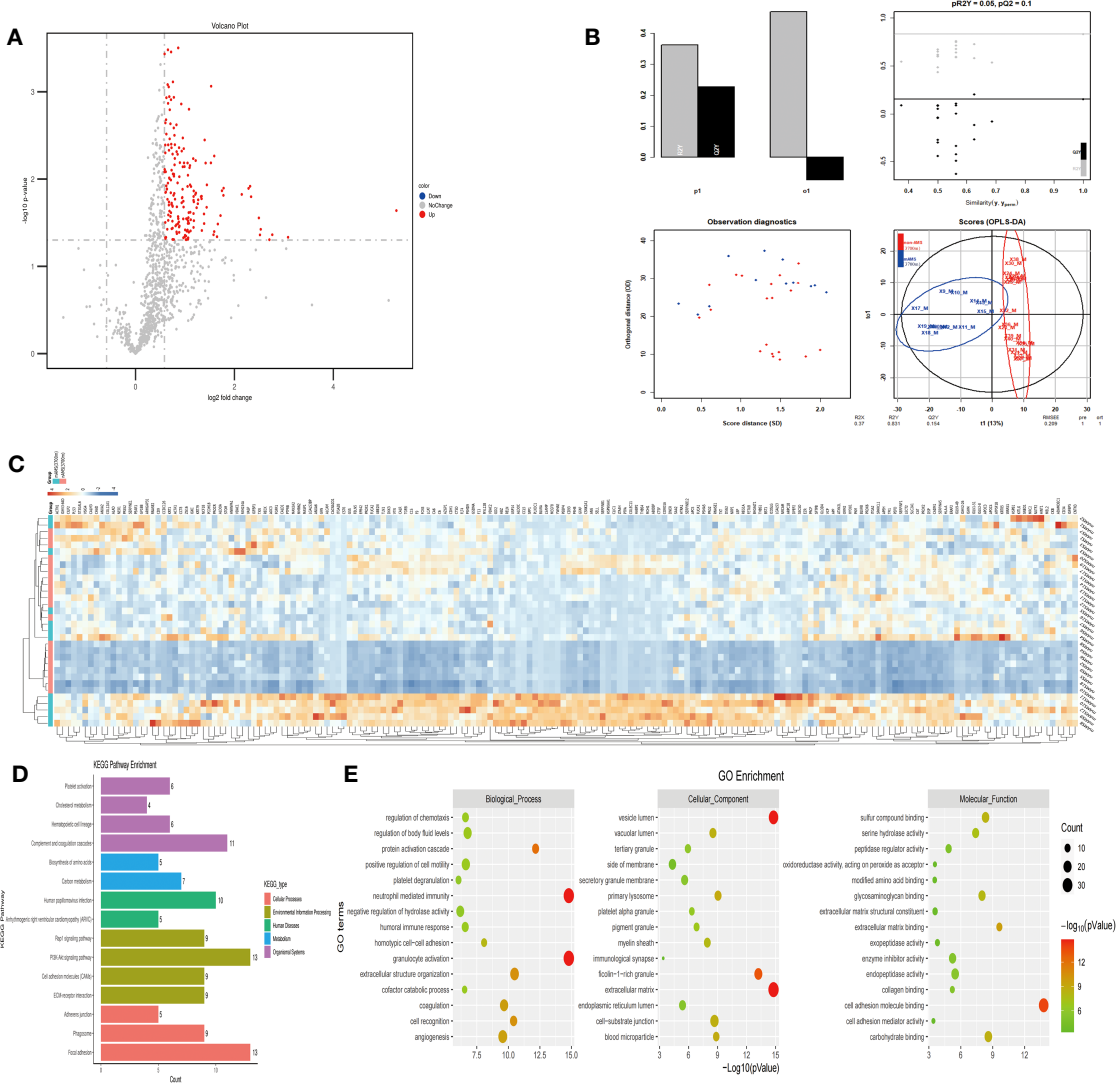
Comparison of plasma differential protein profiles between mAMS group and non-AMS group at the altitude of 3700m. **(A)** Volcano plots show the distribution of DEPs between mAMS group and non-AMS group at the altitude of 3700m. **(B)** PLS-DA plots show clear separation between the mAMS and non-AMS groups at the altitude of 3700m. **(C)** Heatmap represents gene expression trends in mAMS and non-AMS groups at the altitude of 3700m. **(D)** KEGG enrichment shows the canonical pathways of DEPs. **(E)** GO enrichment shows the top fifteen terms in the biological process, cellular component, and molecular function categories between mAMS and non-AMS groups at the altitude of 3700m.

In order to figure out the potential proteins which could predict the AMS susceptibility when individuals further ascend from the middle high-altitude (3700m) to the extreme high-altitude (5000m), we compared between the mAMS group and non-AMS group based on the proteome profiling at the altitude of 3700m. Our proteomic results revealed a total of 183 up-regulated proteins (**Figure 5A**), with a clear classification between mAMS group and non-AMS group by heatmap and PLS-DA analysis (**Figures 5B, C**). GO analysis revealed that the DEPs were mainly associated with the cellular components including vesicle lumen, extracellular matrix and ficolin-1-rich granule, biological processes including neutrophil mediated immunity and granulocyte activation, molecular functions including extracellular matrix binding and cell adhesion molecule binding. KEGG analysis revealed that the enriched pathways mainly included complement and coagulation cascades, phagosome and PI3K-Akt signaling pathway (**Figures 5D, E**).

### 3.4 Identification and validation of selected biomarkers in validation cohort

**Figure 6:**
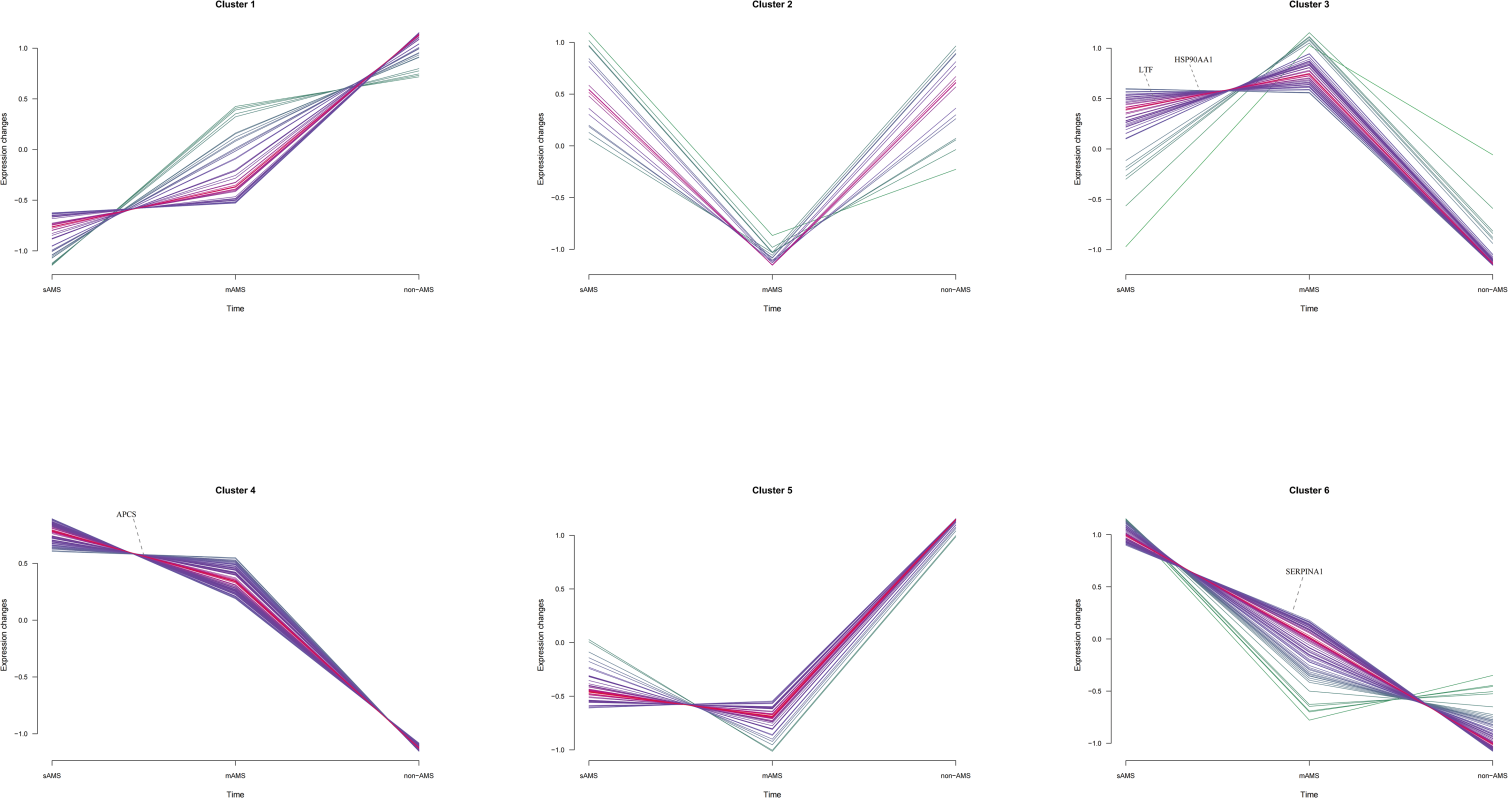
The distribution of SAP, LTF, HSP90-α and AAT in Mfuzz expression pattern clustering with gene names.

Among the potential DEPs in sAMS, mAMS and non-AMS groups at low altitude, we found out four potential biomarkers for predicting AMS which were enriched in inflammatory and immune related terms and had a certain trend among the three groups (**Supplementary Tables S4-6**, **Figure 6**). The four potential biomarkers were HSP90-α, SAP, LTF, AAT. Through clustering Mfuzz expression pattern of all potential DEPs between different groups, HSP90-α and LTF were clustered in cluster 3, SAP was in cluster 4 and AAT was in cluster 6. As showed in **Figure 6**, SAP and AAT were gradually decreased among sAMS group, mAMS group and non-AMS group.

**Figure 7:**
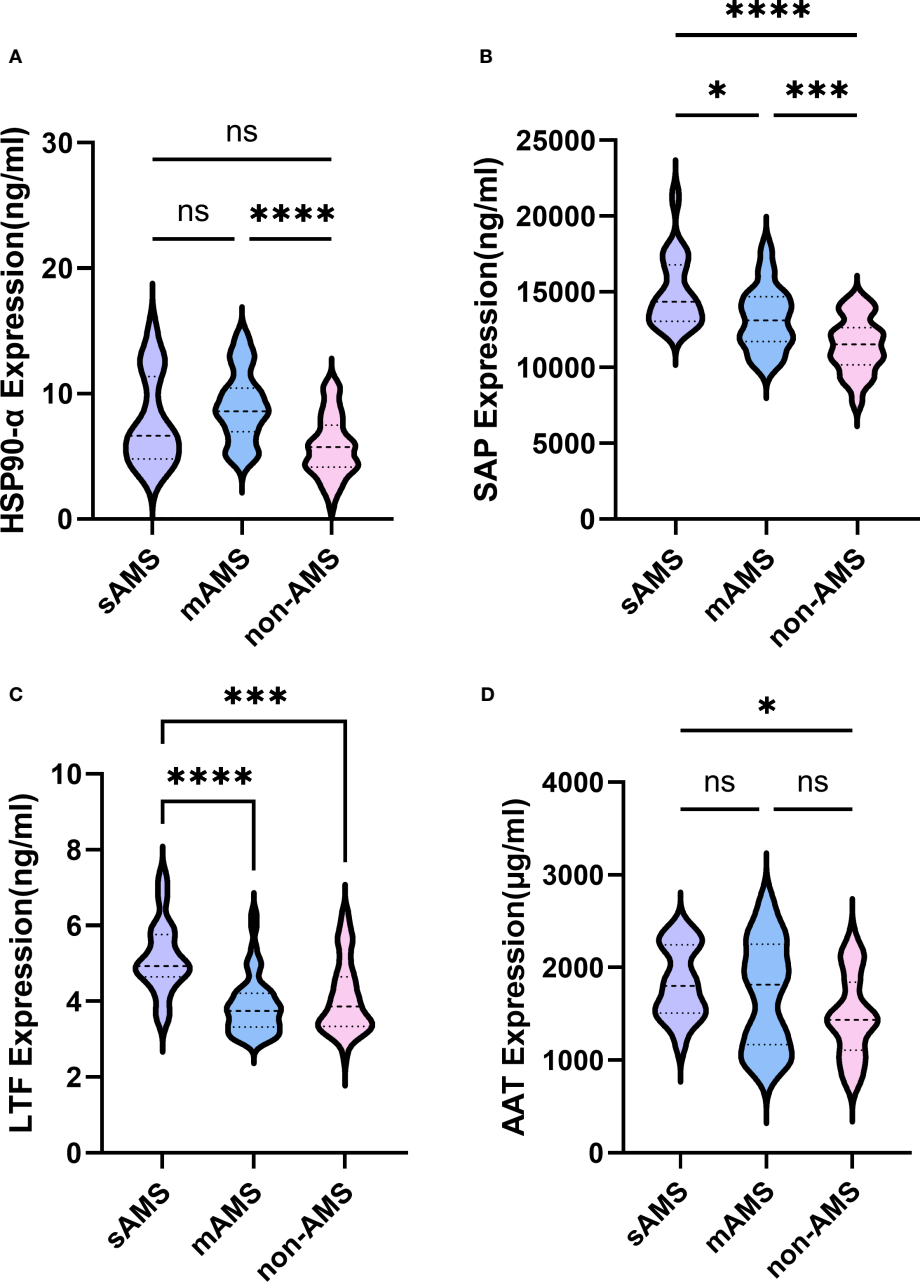
Comparisons of SAP, LTF, HSP90-α and AAT among sAMS, mAMS and non-AMS groups at low altitude. **(A)** The expression of HSP90-α in sAMS, mAMS and non-AMS groups. **(B)** The expression of SAP in sAMS, mAMS and non-AMS groups. **(C)** The expression of LTF in sAMS, mAMS and non-AMS groups. **(D)** The expression of AAT in sAMS, mAMS and non-AMS groups. * *P*<0.05, ***P ≤ 0.001, ****P ≤ 0.0001.

The expression of HSP90-α was statistically higher in mAMS group compared with non-AMS group (*p*<0.0001) (**Figure 7A**). Consistent with the results in discovery cohort, the expression of SAP was statistically higher in sAMS group compared with mAMS (*p*=0.049) and non-AMS group (*p*<0.0001). It was also higher in mAMS group compared with non-AMS group (*p*=0.0003) (**Figure 7B**). The expression of LTF was statistically higher in sAMS group compared with mAMS (*p*<0.0001) and non-AMS group(*p*=0.0005) (**Figure 7C**). The expression of AAT was statistically higher in sAMS group compared with non-AMS group (*p*=0.0191) (**Figure 7D**).

**Figure 8:**
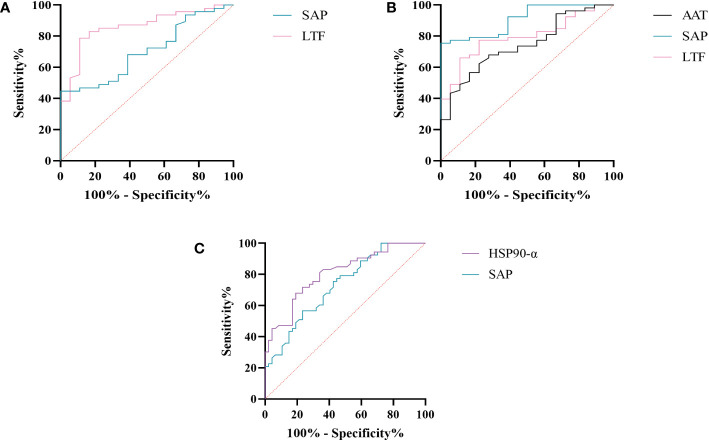
ROC analysis of SAP, LTF, HSP90-α and AAT at low altitude. **(A)** ROC analysis of SAP and LTF for predicting sAMS and mAMS groups. **(B)** ROC analysis of AAT, SAP and LTF for predicting sAMS and non-AMS groups. **(C)** ROC analysis of HSP90-α and SAP for predicting mAMS and non-AMS groups.

**Figure 9:**
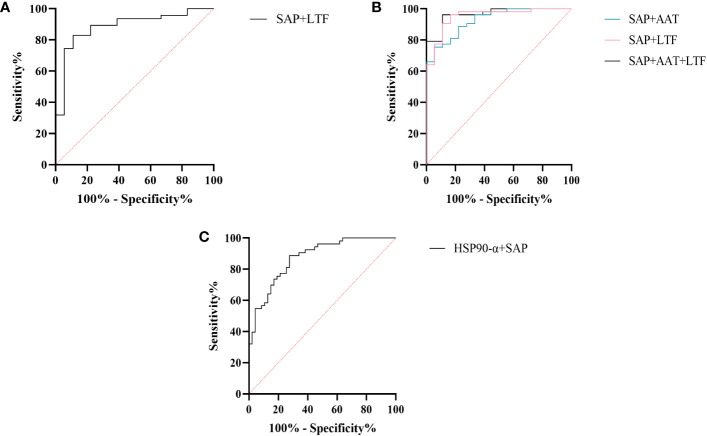
ROC analysis of combined models at low altitude. **(A)** ROC analysis of the combination of SAP and LTF for predicting sAMS and mAMS groups. **(B)** ROC analysis of different combination of AAT, SAP and LTF for predicting sAMS and non-AMS groups. **(C)** ROC analysis of the combination of HSP90-α and SAP for predicting mAMS and non-AMS groups.

We further assessed the evaluation of potential biomarkers at low altitude for predicting sAMS, mAMS, and non-AMS. The expression of SAP and LTF were statistically different between the sAMS group and mAMS group at low altitude. The AUC of SAP for predicting the two groups was 0.704 (95% CI, 0.575 to 0.833, *p*=0.011), with the sensitivity and specificity of 100% and 44.7%. The AUC of LTF was 0.86 (95% CI, 0.764 to 0.956, *p*<0.001), with the sensitivity and specificity of 88.9% and 78.7% (**Figure 8A**, **Table 3**). The combination of SAP and LTF achieved an AUC of 0.887 (95% CI, 0.796 to 977, *p*<0.001), with the sensitivity and specificity of 75.5% and 94.4% (**Figure 9A**, **Table 4**).

**Table 3:** Characteristics of potential predictive proteins for ROC analysis.

	Measure	AUC	95%CI	Sensitivity (%)	Specificity (%)	Cutoff value (ng/mL) (μg/mL)*	*P* value
sAMS - mAMS	SAP	0.704	0.575 to 0.833	100	44.7	12606.5786	0.011
	LTF	0.86	0.764 to 0.956	88.9	78.7	4.2409	<0.001
sAMS - non-AMS	AAT	0.744	0.623 to 0.866	77.8	62.3	1507.4295*	0.002
	SAP	0.908	0.84 to 0.975	100	75.5	12598.7226	<0.001
	LTF	0.794	0.687 to 0.900	77.8	77.4	4.7085	<0.001
mAMS - non-AMS	HSP90-α	0.805	0.721 to 0.889	80.9	67.9	6.3207	<0.001
	SAP	0.728	0.631 to 0.825	76.6	56.6	11695.6447	<0.001

The symbol * means that the unit of cutoff value is μg/ml.

**Table 4:** Characteristics of predictive models for ROC analysis.

	Measures	AUC	95%CI	Sensitivity (%)	Specificity (%)	*P* value
sAMS - mAMS	SAP+LTF	0.887	0.796 to 0.977	83	88.9	<0.001
sAMS - non-AMS	SAP+AAT	0.927	0.866 to 0.987	75.5	94.4	<0.001
	SAP+LTF	0.951	0.898 to 1.000	90.6	88.9	<0.001
	SAP+AAT+LTF	0.965	0.927 to 1.000	96.2	88.9	<0.001
mAMS - non-AMS	HSP90-α+SAP	0.874	0.807 to 0.940	88.7	72.3	<0.001

The expression of AAT, SAP and LTF were statistically different between the sAMS group and non-AMS group at low altitude. The AUC of AAT for predicting the two groups was 0.744 (95% CI, 0.623 to 0.866, p=0.002), with sensitivity and specificity of 77.8% and 62.3%. The AUC of SAP was 0.908 (95% CI, 0.84 to 0.975, *p*<0.001), with sensitivity and specificity of 100% and 75.5%. The AUC of LTF was 0.794 (95% CI, 0.687 to 0.9, p<0.001), with sensitivity and specificity of 77.8% and 77.4% (**Figure 8B**, **Table 3**). The combination of AAT, SAP and LTF achieved an AUC of 0.965 (95% CI, 0.927 to 1, *p*<0.001), with sensitivity and specificity of 96.2% and 88.9% (**Figure 9B**, **Table 4**).

The expression of HSP90-α and SAP were statistically different between the mAMS group and non-AMS group at low altitude. The AUC of HSP90-α for predicting the two groups was 0.805 (95% CI, 0.721 to 0.889, *p*=0.002), with sensitivity and specificity of 80.9% and 67.9%. The AUC of SAP was 0.728 (95% CI, 631 to 0.825, *p*<0.001), with sensitivity and specificity of 76.6% and 56.6% (**Figure 8C**, **Table 3**). The combination of HSP90-α and SAP achieved an AUC of 0.874(95% CI, 0.807 to 0.94, *p*<0.001), with sensitivity and specificity of 88.7% and 72.3% (**Figure 9C**, **Table 4**).

## 4 Discussion

Our current study investigated the plasma proteome differences between AMS-susceptible and AMS-resistant individuals at low altitude, aiming to identify potential biomarkers for screening AMS-susceptible individuals. To our knowledge, this investigation is the first to comprehensively assess the plasma proteome at one low altitude(1400m) and two high altitudes (3700m and 5000m). Furthermore, we categorized AMS-susceptible individuals into severe and moderate subgroups based on their different reaction at two high altitudes. Our study on the plasma proteome of Chinese volunteers from lowland provides novel insights into variation pattern of plasma proteome between mAMS and sAMS individuals, between mAMS and non-AMS individuals, between sAMS and non-AMS individuals. We identified potential immune and inflammatory response related biomarkers at low altitude for the prediction of sAMS, mAMS and non-AMS individuals. Then we further assessed and quantified their plasma concentration in these three groups at low altitude, revealing that SAP, LTF, HSP90-α, AAT exhibit discriminatory potential among these groups at low altitude.

In our current study, plasma samples of 40 healthy volunteers were utilized for proteomic analysis. After comprehensive categorization, we provided in-depth insights about AMS susceptibility of lowlanders and prediction for middle high-altitude (around 3500m) and extreme high-altitude (around 5000m). We then compared the plasma proteome between sAMS group and non-AMS group, between mAMS group and non-AMS group. The DEPs were 248 and 228, respectively and were enriched in the common biological pathways including granulocyte activation, neutrophil mediated immunity, humoral immune response (**Supplementary Tables S4, S5**). Comparison between sAMS group and mAMS group found only 23 DEPs, which were also enriched in several biological pathways, such as neutrophil mediated immunity and granulocyte activation (**Supplementary Table S6**). DEPs including SAP, LTF, HSP90-α, AAT were enriched in these above-mentioned pathways, and were further screened out as candidate biomarkers for distinguishing AMS-susceptible individuals from AMS-resistant individuals.

SAP is an inflammatory response glycoprotein. Its gene name is called APCS. As a component of the innate immune response, SAP can be produced by cytokines stimulation (15, 16). The administration of SAP induces the production of IL-10 while suppressing the differentiation of M2a macrophages and promoting the generation of immuno-regulatory macrophages (17, 18). Studies have identified SAP to be associated with hypoxia-related diseases. SAP may be a marker for the development of atherosclerosis and/or the progression of angina pectoris and myocardial infarction (19). The inflammation characterized by elevated levels of SAP in plasma exhibits a more pronounced manifestation following myocardial infarction (20). In the post-acute myocardial infarction(AMI) phase, there is a decrease in immune response-inflammatory proteins, except for SAP which demonstrates an elevation associated with activation of classical complement pathway (21). The expression of SAP is elevated in the acute period of HAPE patients’ plasma compared to the recovery period (20). In this study, SAP was found to be enriched in immune and inflammatory related pathways between AMS susceptible and AMS resistant groups through plasma proteomics at low altitude. Interestingly, the results of plasma proteome showed that SAP was gradually decreased among sAMS group, mAMS group and non-AMS group. Furthermore, we validated that the serum concentration of SAP differed between any two groups. The AUC of SAP could reach 0.908 when predicting sAMS and non-AMS groups. The AUC could reach 0.965 after combination of SAP with AAT and LTF, with sensitivity and specificity of 96.2% and 88.9%, suggesting that SAP may be a candidate biomarker for prediction of AMS.

Heat shock protein 90AA1(HSP90AA1), encoding HSP90-α, belongs to the heat shock protein family which is involved in immune and inflammatory responses in a variety of diseases (22–24). HSP90AA1 exhibits potential as secreted extracellular factors involved in the processes of wound healing and inflammation (25). Qian et al. found that HSP90AA1 was implicated in immune system dysfunction, an early event of Alzheimer’s disease (26). In addition, HSP90 pathway plays a role in human adaptation to high altitude as a molecular mechanism of the hypoxic response (27). Zinc finger function, which is impaired in high-altitude-adapted Tibetans, could recruit PHD2, a key oxygen sensor, to the HSP90 pathway for facilitating hydroxylation of HIF-α. The ablation of zinc finger function resulted in upregulation of the erythropoietin gene, leading to erythrocytosis, and an enhanced hypoxic ventilatory response (27). In our current study, we observed a significant difference in HSP90-α levels between mAMS group and non-AMS group at low altitude. The AUC of HSP90-α could reach 0.805 when predicting mAMS and non-AMS groups. The AUC could reach 0.874 after combination of HSP90-α with SAP, with sensitivity and specificity of 88.7% and 72.3%. These findings suggest that HSP90-α may have potential as a distinguishing biomarker.

AAT, an acute phase protein produced primarily by the liver and encoded by SERPINA1 gene, acts as a plasma serine protease inhibitor to mitigate protease-mediated tissue damage during inflammation (28). On the other hand, AAT exhibits elevated plasma concentrations in response to inflammatory stimuli. Takei N et al. discovered that patients of chronic obstructive pulmonary disease(COPD) who had higher serum AAT levels experienced accelerated lung function decline (29). Previous study found that AAT levels were mildly elevated in individuals prone to HAPE during the initial phase of hypobaric hypoxia exposure (30). However, the concentration of AAT in the serum was elevated in highland natives compared to those living at sea level, which suggested that AAT may facilitate adaptation to the hypobaric hypoxia environment of high altitude (31). Nevertheless, it should be noted that the actions of AAT varied across different diseases as reported by previous studies. Given its multifunctionality, the precise involvement of AAT in AMS remains elusive. In this study, we found that AAT levels were mildly higher in sAMS group compared to non-AMS group. However, further investigations with an expanded sample size are warranted.

LTF, or Lactoferrin (LF), is an 80kDa iron-binding glycoprotein (32). It was found that yak milk, a major food resource necessary for highland natives to cope with the challenges of altitude, has a higher LTF content than cow milk, suggesting that LTF may facilitate adaptation to the highland environment (33). As a crucial defense molecule against infection, LTF-containing immune complexes was related to triggering activation of monocytes and macrophages, which may further influence inflammatory related pathways and cytokines (34). Granulocyte colony-stimulating factor (G-CSF) induces the upregulation of LTF expression in secondary granules of neutrophils (35). LTF promotes macrophage activation through TLR4-dependent and TLR4-independent pathways, suggesting an important role in immunomodulation (35, 36). We found that the expression of LTF differed between AMS susceptible and resistant groups at low altitude, especially between sAMS group and mAMS group, and between sAMS group and non-AMS group. The AUC of LTF could reach 0.86 when predicting sAMS and mAMS groups, which could reach 0.887 after combination of SAP, with sensitivity and specificity of 83% and 88.9%. As a multifaceted protein, LTF may play an important role of immune modulation in AMS and be a potentially predictive biomarker.

In conclusion, our present study demonstrates significant differential expression of biological pathways involved in granulocyte activation, neutrophil mediated immunity, humoral immune response between AMS-susceptible and AMS-resistant individuals at low altitude through plasma proteome. Furthermore, DEPs including SAP, LTF, HSP90-α, AAT could be considered as potential biomarkers for distinguishing AMS-resistant and AMS-susceptible individuals at low altitude for AMS prediction. More clinical samples and further mechanism study are still required for investigation of potential effect of these pathways and DEPs in the development of AMS.

## Data availability statement

The datasets presented in this study can be found in online repositories. The names of the repository/repositories and accession number(s) can be found below: https://proteomecentral.proteomexchange.org/, PXD045131.

## Ethics statement

The studies involving humans were approved by the Ethic Committee of Chinese PLA General Hospital. The studies were conducted in accordance with the local legislation and institutional requirements. The participants provided their written informed consent to participate in this study.

## Author contributions

HG performed the experiments, analyzed data, and wrote the manuscript. QW collected samples, assisted with experiments performation, writing and editing. TL, JC, CZ and WS recruited volunteers, distributed and returned questionnaires and assisted with sample collection. YX, QC and HL evaluated LLS of participants and conducted sample processing. RH participated in collecting samples in high altitude field experiments. CW and CBW designed the study, and reviewed the manuscript. All authors contributed to the article and approved the submitted version.
