# Supplementary material for: Potential plasma biomarkers at low altitude for prediction of acute mountain sickness
Source: Front Immunol. 2023 Sep 28;14:1237465. doi: 10.3389/fimmu.2023.1237465 (PMC10569122; doi:10.3389/fimmu.2023.1237465)
Supplement: Supplementary file 1 [file DataSheet_1.docx]

**Supplementary Table S1 Lake Louise Acute Mountain Sickness Score**

| **Headache** |
| --- |
| 0-The symptom of headache is not obvious, without painful expression, does not affect daily activities. |
| 1-The symptom of headache is mild, with painful expression, significantly improved after individuals taking general painkillers, and does not affect daily activities. |
| 2-The symptom of headache is severe, with painful expression, relieved after individuals taking general painkillers, and affects daily activities. |
| 3-The symptom of headache is severe and can not be tolerated.Individuals may bedridden and taking general painkillers is ineffective. |
| **Gastrointestinal symptoms** |
| 0-Good appetite. |
| 1-Poor appetite or nausea. |
| 2-Moderate nausea or vomiting. |
| 3-Severe nausea and vomiting, incapacitating. |
| **Fatigue and/or weakness** |
| 0-Not tired or weak. |
| 1-Mild fatigue/weakness. |
| 2-Moderate fatigue/weakness. |
| 3-Severe fatigue/weakness. |
| **Dizziness** |
| 0-No dizziness. |
| 1-Mild dizziness. |
| 2-Moderate dizziness. |
| 3-Severe dizziness, incapacitating. |

**Supplementary Table S2 Demographic information, AMS symptoms and LLS of discovery cohort at altitude of 5000m**

|  | AMS group (n=20) | Non-AMS group (n=20) | *P* |
| --- | --- | --- | --- |
| Age | 23.5(22.25 , 26.75) | 22(21 , 25) | 0.094 |
| Weight(kg) | 69.6(7.12593) | 69.3(6.0793) | 0.887 |
| Height(cm) | 175.15(5.37318) | 175.05(3.89973) | 0.947 |
| BMI | 22.6704(1.83082) | 22.636(2.1183) | 0.957 |
| Smoking | 60%（12/20） | 60%(12/20) | 1.000 |
| Headache | 100%（20/20） | 35%（7/20） | <0.001 |
| Gastrointestinal upset | 75%（15/20） | 30%（6/20） | 0.004 |
| Fatigue | 100%（20/20） | 65%（13/20） | 0.008 |
| Dizziness | 95%（19/20） | 45%（9/20） | 0.002 |
| Insomnia | 100%（20/20） | 45%（9/20） | <0.001 |
| LLS | 5(4 , 5.75) | 2(1.25 , 2) | <0.001 |

**Supplementary Table S3 Demographic information, AMS symptoms and LLS of discovery cohort at altitude of 3700m**

|  | AMS group (n=8) | Non-AMS group (n=32) | *P* |
| --- | --- | --- | --- |
| Age | 23.5(22.25,27.75) | 23(21 , 25.75) | 0.332 |
| Weight(kg) | 71.75(8.77903) | 68.875(5.89012) | 0.272 |
| Height(cm) | 177.375(3.81491) | 174.5313(4.69718) | 0.122 |
| BMI | 23.8724(20.4753 , 24.6825) | 22.6616(21.6391 , 23.557) | 0.379 |
| Smoking | 50%（4/8） | 62.5%（20/32） | 0.69 |
| Headache | 100%（8/8） | 0（0/32） | <0.001 |
| Gastrointestinal upset | 87.5%（7/8） | 3.1%（1/32） | <0.001 |
| Fatigue | 100%（8/8） | 56.3%（18/32） | 0.034 |
| Dizziness | 75%（6/8） | 28.1%（9/32） | 0.036 |
| Insomnia | 75%（6/8） | 81.3%（26/32） | 0.65 |
| LLS | 4(3,4) | 1(0,2) | <0.001 |

**Supplementary Table S4 The biological pathways in GO analysis of the plasma proteome between sAMS group and non-AMS group at low altitude**

| GeneSet | Description | *p*Value | FDR | UserId |
| --- | --- | --- | --- | --- |
| GO:0052547 | regulation of  peptidase activity | 1.47306E-07 | 6.33029E-06 | P01031;Q14624;P01008; P01009;Q16610;P04004; P08697;P12111;P05543; P04406;Q9UK55;P02788; P08185;P01034;P62258; Q13103;P01033;Q99497; O95980;P07203;P03973;P12931 |
| GO:0006898 | receptor-mediated  endocytosis | 3.21714E-08 | 1.54518E-06 | P04114;P02787;P05156; P04004;P00738;P00739; Q08380;P22897;Q8NBP7; P02654;P61769;P02655; A1L4H1;P09486;P05106; Q01518;P07900;Q9Y4L1;Q00610 |
| GO:0051604 | protein  maturation | 7.25256E-09 | 3.82046E-07 | P00734;P01031;P00751; P04003;P13671;P00736; P05156;P04004;P08697; P15169;P05452;Q03591; P00740;P07360;Q9BXR6; Q8NBP7;P25774;Q9UBR2; P13987;P08174;P12931 |
| GO:0050878 | regulation of  body fluid levels | 3.13735E-10 | 2.04932E-08 | P00747;P00734;P01008; P12259;P01009;P04264; P21333;P04004;P08697; P00488;P00740;P08779; P00742;P18206;Q9UK55; P22891;P08514;P05106; P19021;P08581;P17301; P13987;P16109;P47756; P08123;P20930;P04899;P12931 |
| GO:0050817 | coagulation | 3.07116E-10 | 2.04932E-08 | P00747;P00734;P01008; P12259;P01009;P04264; P21333;P04004;P08697; P00488;P00740;P00742; P18206;Q9UK55;P22891; P08514;P05106;P17301; P13987;P16109;P47756; P08123;P12931 |
| GO:0051346 | negative regulation  of hydrolase activity | 1.19134E-10 | 9.26411E-09 | P01031;Q14624;P01008; P01009;Q16610;P04004; P08697;P12111;P05543; P04406;Q9UK55;P02788; P08185;P02743;P02654; P01034;P62258;Q13103; Q9Y5C1;P01033;Q99497; O95980;Q15257;P07203; P03973;P04899;P12931 |
| GO:0050727 | regulation of  inflammatory response | 7.36574E-11 | 6.01413E-09 | P00734;P01031;P00751; P04003;P13671;P04264; P00736;P05156;P04004; Q96PD5;P15169;Q03591; P07360;P33151;Q9BXR6; Q13093;P02743;P00533; P40189;Q10588;P17301; Q99497;P13987;P08174;P07203 |
| GO:0043062 | extracellular structure  organization | 3.79108E-12 | 3.43935E-10 | P04114;P00747;P04004; P08697;P12111;P24821; P02766;Q13093;P08514; P55058;P02654;P27487; P02655;P01034;P09486; P05106;P25774;Q6UY14; P17813;P01033;Q86TH1; P17301;O95980;P07478; P08123;P13497;P35613;P10451 |
| GO:0045861 | negative regulation  of proteolysis | 5.26246E-14 | 7.16133E-12 | P00734;P01031;P04003; Q14624;P01008;P01009; Q16610;P04004;P08697; P12111;P05543;P04406; Q9UK55;P02788;P08185; P01034;P13716;P62258; Q13103;P01033;Q99497; Q9UBR2;P13987;O95980; P08174;P07203;P03973;P12931 |
| GO:0006959 | humoral immune  response | 1.11022E-15 | 1.64818E-13 | P00734;P01031;P00751; P04003;P13671;P04264; P00736;P05156;P04004; Q96PD5;P15169;Q03591; P04406;P07360;P02788; Q9BXR6;P00746;P02743; B9A064;Q10588;P13987; P08174;P07478;P59665; P03973;P10645 |
| GO:0072376 | protein activation  cascade | 0 | 0 | P00734;P01031;P00751; P04003;P13671;P01008; P04264;P00736;P05156; P04004;P00488;P15169; Q03591;P00740;P00742; P07360;Q9BXR6;P00746; P02743;B9A064;P13987;P08174 |
| GO:0002576 | platelet  degranulation | 0 | 0 | P00747;P02787;Q14624; P12259;P01009;P21333; Q16610;P08697;P00488; P05452;O00391;Q08380; P18206;P00746;P04075; P08514;P02763;P09486; P05019;P05106;P49908; Q13103;P01033;P16109 |
| GO:0002526 | acute inflammatory  response | 0 | 0 | P00734;P01031;P00751; P04003;Q14624;P13671; P01008;P01009;P00736; P05156;P04004;P00738; P08697;P15169;P00739; Q03591;P07360;Q9BXR6; P02743;P40189;P02763; Q99497;P13987;P08174;P08887 |
| GO:0002446 | neutrophil mediated  immunity | 0 | 0 | P00734;P01009;P04264; P00738;P04040;O00391; P15144;P02766;P18206; P02788;P00746;P04075; Q92820;P14923;Q02413; P14151;P00491;P61769; P01034;P02763;P13716; Q10588;P50395;Q9NZK5; P25774;P07437;P07339; Q08554;Q01518;P07900; P27105;Q9UBR2;Q9NZT1; P13987;P08174;P07478; P13727;P02792;P59665; P28676;P03973;P53396; O00584;Q70J99;Q14974 |
| GO:0036230 | granulocyte  activation | 0 | 0 | P01009;P04264;P00738; P04040;O00391;P15144; P02766;P18206;P02788; P00746;P04075;Q92820; P14923;Q02413;P14151; P00491;P61769;P01034; P02763;P13716;Q10588; P50395;Q9NZK5;P25774; P07437;P07339;Q08554; Q01518;P07900;P27105; Q9UBR2;Q9NZT1;P13987; P08174;P07478;P13727; P02792;P59665;P28676; P03973;P53396;O00584; Q70J99;Q14974 |

**Supplementary Table S5 The biological pathways in GO analysis of the plasma proteome between mAMS group and non-AMS group at low altitude**

| GeneSet | Description | *p*Value | FDR | UserId |
| --- | --- | --- | --- | --- |
| GO:0034367 | protein-containing  complex remodeling | 8.16638E-08 | 4.59852E-06 | P04114;P02656;Q13093; P11597;P55058;P02654; P06858 |
| GO:0072593 | reactive oxygen species  metabolic process | 5.93676E-08 | 3.46241E-06 | P00734;P07996;P00738; P69905;P68871;P04040; P02042;P32119;P69892; P00533;Q10588;P07900; Q99497;P30041;P37840; P11413;P04899 |
| GO:0051346 | negative regulation of  hydrolase activity | 1.60929E-08 | 1.1426E-06 | P01009;P07996;P04004; P12111;P05543;P04406; P02788;P02656;P02743; P02654;P01034;P30740; P62258;Q13103;P01033; Q99497;P37840;O95980; Q15257;P20810;P54578; P04899;P12931 |
| GO:0050727 | regulation of  inflammatory response | 1.59672E-08 | 1.1426E-06 | P00734;P04003;P04264; P00736;P04004;Q96PD5; P15169;Q9BXR6;Q13093; P02743;P00533;P40189; Q10588;P17301;Q99497; P37840;P13987;P08174; P06858;O75326;P01130 |
| GO:0051187 | cofactor catabolic  process | 9.77862E-09 | 7.60404E-07 | P00738;P69905;P68871; P04040;P02042;P32119; P69892;P30043;P30041; P37840 |
| GO:0016999 | antibiotic metabolic  process | 8.42046E-09 | 6.8753E-07 | P00738;P69905;P68871; P04040;P02042;P32119; P69892;P00533;Q99497; P30041;P37840;P40925; P10768;P08319 |
| GO:0072376 | protein activation  cascade | 3.23934E-09 | 2.78412E-07 | P00734;P04003;P04264; P00736;P04004;P15169; P00740;Q9BXR6;P02743; B9A064;P13987;P08174 |
| GO:0097006 | regulation of plasma  lipoprotein particle levels | 1.27456E-09 | 1.15631E-07 | P04114;P80108;P02656; Q13093;Q8NBP7;P11597; P55058;P02654;P07355; P07237;P06858;P01130 |
| GO:0006959 | humoral immune  response | 8.18383E-11 | 1.02802E-08 | P00734;P04003;P04264; P00736;P04004;Q96PD5; P15169;P04406;P02788; Q9BXR6;P02743;Q02383; B9A064;P61626;Q10588; P13987;P08174;P07478; P81605;Q9H1E1 |
| GO:0006898 | receptor-mediated  endocytosis | 3.88201E-11 | 5.28276E-09 | P04114;P04004;P00738; P69905;P68871;P00739; Q08380;O14791;P02656; P22897;Q8NBP7;P02654; P61769;A1L4H1;P09486; P07355;Q01518;P07900; P07307;P37840;P01130; Q8WWQ8 |
| GO:0043062 | extracellular  structure organization | 2.41939E-11 | 3.59169E-09 | P04114;P07996;P04004; P12111;P24821;P02766; P02656;Q13093;P11597; P55058;P02654;P01034; P09486;P07355;P25774; P07237;P01033;Q86TH1; P17301;O95980;P07478; P08123;P13611;P06858; O94769;P10451 |
| GO:0002526 | acute inflammatory  response | 2.38414E-11 | 3.59169E-09 | P00734;P04003;P01009; P00736;P04004;P00738; P15169;P00739;Q9BXR6; P02743;O95497;P0DJI9; P40189;Q99497;P13987; P08174;P08887 |
| GO:0045861 | negative regulation  of proteolysis | 2.11371E-11 | 3.59169E-09 | P00734;P04003;P01009; P07996;P04004;P12111; P05543;P04406;P02788; P01034;P30740;P13716; P62258;Q13103;P01033; Q99497;P37840;Q9UBR2; P13987;O95980;P08174; P20810;P54578;P12931 |
| GO:0036230 | granulocyte  activation | 0 | 0 | P01009;P04264;P00738; P68871;P04040;O00391; P02766;P18206;P02788; P14151;O95497;P61626; P61769;P01034;P30740; P13716;Q10588;P50395; P54108;P07355;P25774; P07437;P07339;Q08554; Q01518;P07900;P27105; P30041;Q9UBR2;P13987; P08174;P07478;P02792; P15309;Q15833;Q70J99; Q14974 |
| GO:0002446 | neutrophil mediated  immunity | 0 | 0 | P00734;P01009;P04264; P00738;P68871;P04040; O00391;P02766;P18206; P02788;P14151;O95497; P61626;P61769;P01034; O75083;P30740;P13716; Q10588;P50395;P54108; P07355;P25774;P07437; P07339;Q08554;Q01518; P07900;P27105;P30041; Q9UBR2;P13987;P08174; P07478;P02792;P15309; Q15833;Q70J99;Q14974 |

**Supplementary Table S6 The biological pathways in GO analysis of the plasma proteome between sAMS group and mAMS group at low altitude**

| GeneSet | Description | pValue | FDR | UserId |
| --- | --- | --- | --- | --- |
| GO:1902903 | regulation of supramolecular fiber organization | 0.007285359 | 0.645846145 | P07737;P31146;P01130 |
| GO:0007159 | leukocyte cell-cell adhesion | 0.006691411 | 0.645846145 | P04083;P31146;Q14623 |
| GO:0010721 | negative regulation of cell development | 0.006691411 | 0.645846145 | Q9UM47;P01130;O75164 |
| GO:0050867 | positive regulation of cell activation | 0.006182701 | 0.645846145 | P04083;P31146;Q14623 |
| GO:0051961 | negative regulation of nervous system development | 0.005490447 | 0.645846145 | Q9UM47;P01130;O75164 |
| GO:0070661 | leukocyte proliferation | 0.004706079 | 0.645846145 | P04083;P31146;Q14623 |
| GO:0042063 | gliogenesis | 0.003994386 | 0.645846145 | P04083;P01130;O75164 |
| GO:0031016 | pancreas development | 0.003993878 | 0.645846145 | P04083;Q14623 |
| GO:0006081 | cellular aldehyde metabolic process | 0.003694924 | 0.645846145 | O95336;P08319 |
| GO:0036230 | granulocyte activation | 0.002911589 | 0.645846145 | P00746;Q9NZT1;P03973;O00584 |
| GO:0002446 | neutrophil mediated immunity | 0.002828241 | 0.645846145 | P00746;Q9NZT1;P03973;O00584 |
| GO:0002694 | regulation of leukocyte activation | 0.002530384 | 0.645846145 | P04083;P31146;P01130;Q14623 |
| GO:0018149 | peptide cross-linking | 0.00244646 | 0.645846145 | P04083;Q08188 |
| GO:0008544 | epidermis development | 0.002066897 | 0.645846145 | P48668;P04083;Q9NZT1;Q08188 |
| GO:0050866 | negative regulation of cell activation | 0.001309834 | 0.645846145 | P04083;P01130;Q14623 |
